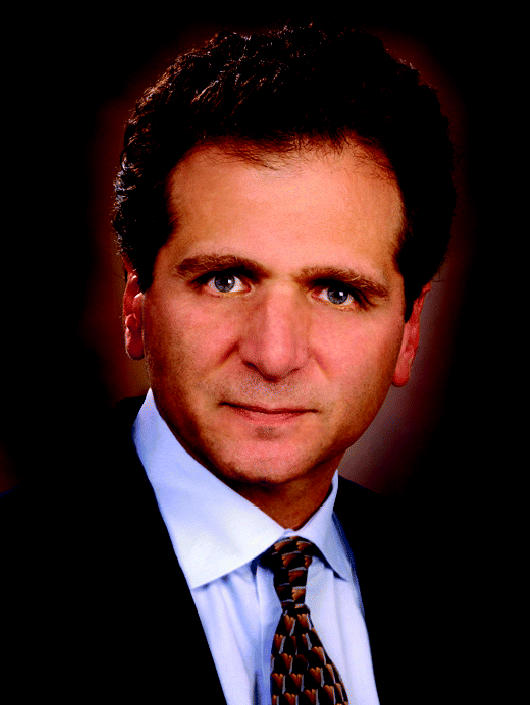# Schwartz Named New NIEHS/NTP Director

**Published:** 2004-12

On 25 October 2004 NIH director Elias Zerhouni announced the appointment of David Schwartz as the new director of the NIEHS and the National Toxicology Program. Schwartz, who will assume his new duties on 4 April 2005, is currently director of the Pulmonary, Allergy, and Critical Care Division and vice chair of research in the Department of Medicine at Duke University. While at Duke, Schwartz has also played a leading role in developing interdisciplinary Centers in Environmental Health Sciences, Environmental Genomics, and Environmental Asthma. Schwartz’s research has focused on the genetic and biological determinants of environmental lung disease and host defense.

Schwartz is filling the position left open by Kenneth Olden, who stepped down from the post late in 2003, but who agreed to remain in the position until his successor was named. Olden will stay on at the NIEHS as a researcher in the intramural program.

At the announcement of the appointment, DHHS secretary Tommy Thompson called Schwartz “one of the nation’s outstanding researchers in environmental health.” Thompson and Zerhouni acknowledged the leadership role that Schwartz is taking on at the NIEHS as environmental factors are being implicated more often in the etiology of disease. Zerhouni touted Schwartz’s interdisciplinary approach as one that “will help lead us to well-conceived strategies for preventing, diagnosing, and treating disease.”

As director of the NIEHS, Schwartz will oversee a $711 million budget that funds multidisciplinary biomedical research programs, as well as prevention and intervention efforts that encompass training, education, technology transfer, and community outreach. The NIEHS currently supports more than 850 research grants.

“I am delighted and honored to join NIH,” said Schwartz. “My vision for NIEHS is to improve human health by supporting integrated research and career development in environmental sciences, environmental medicine, and environmental public health. Given recent advances in biomedical research and computational biology, NIEHS is well positioned to use its expertise in toxicology to understand human biology, disease pathogenesis, and the unique distribution of disease in different populations.”

## Figures and Tables

**Figure:**